# Awareness, Utilization and Health Outcomes of National Essential Public Health Service Among Migrants in China

**DOI:** 10.3389/fpubh.2022.936275

**Published:** 2022-07-11

**Authors:** Xinpeng Xu, Qinglong Zhang, Hua You, Qifeng Wu

**Affiliations:** ^1^School of Public Health, Nanjing Medical University, Nanjing, China; ^2^Institute of Healthy Jiangsu Development, Nanjing Medical University, Nanjing, China; ^3^Jiangsu Health Development Research Center, Nanjing, China

**Keywords:** awareness, utilization, national essential public health service, health, migrants

## Abstract

**Background:**

The national essential public health service (NEPHS) has been in operation for more than a decade. Numerous studies examined the utilization of NEPHS by migrants and the factors that influence it, but few examined the effect of NEPHS awareness and utilization on the health of inhabitants, particularly migrants. The purpose of this study is to ascertain the level of awareness and utilization of NEPHS, as well as to examine their health-improving effects on migrants.

**Methods:**

Based on the data from the 2017 China Migrants Dynamic Survey, linear probability model, ordered logit model and the propensity score matching methods were employed to investigate impact of awareness and utilization of NEPHS on the health among Chinese migrants. Mediating effect model were used to identify the mechanism of the impact of NEPHS on health.

**Results:**

The findings indicated that migrants' awareness and utilization of NEPHS are still insufficient. After adjusting for other factors, the study discovered that increased awareness and use of NEPHS had a beneficial influence on migrants' self-rated health. Further heterogeneity analysis revealed significant disparities in the health consequences of NEPHS awareness and utilization across subgroups. The effect of increased awareness and usage of NEPHS on health is stronger for middle-aged and elderly people, women, and low-educated migrants with urban household registration. The estimated results of the mediating effect model supported the mechanism that increased NEPHS awareness among the floating population could encourage its utilization and further improve the floating population's health.

**Conclusions:**

Given that migrants' NEPHS utilization is still low and that NEPHS utilization has a positive effect on health, some targeted strategies, such as a variety of new media communication methods, health education related to occupational disease and tuberculosis prevention, and targeted NEPHS projects for specific groups, such as men, young and middle-aged groups, those with a high level of education, and rural migrants, should be conducted to improve the health of migrants.

## Background

The country's urbanization and economic development have benefited significantly from the floating population (1, 2). Sustaining economic and social progress has also facilitated population movement (3). China's entire migrant population has been expanding over the last few decades. According to the 7th National Population Census, China's migrant population has surpassed 370 million, a 69.73 percent growth over 2010 (4). The health of this population is jeopardized by high occupational health risks and exposure to substandard living conditions (5, 6), and the existence of the household registration system places the floating population at a significant disadvantage in terms of social welfare and employment opportunities when compared to local residents (7, 8). Additionally, it denies them the same access to healthcare as local residents (9, 10).

To effectively protect the floating population's right to health, China has developed a number of health regulations for migrants in China, and clear arrangements have been established to improve this population's fundamental public health service (11). China began implementing national essential public health services (NEPHS) in 2009; In 2013, the National Health and Family Planning Commission issued *Guidance on the implementation of pilot projects for the administration and equalization of public health services and family planning for migrants*, and the pilot to provide equal access to NEPHS for migrants was launched. To more effectively promote equal access to basic public health services for migrants, the National Health and Family Planning Commission, in collaboration with relevant departments, issued *Guidance on the management of basic public health services and family planning for migrants* in 2014. The guidance emphasized the importance of prioritizing the implementation of six basic public health services for migrants, including child vaccination, prevention and control of infectious diseases, maternal and child health care, health records, family planning and health education (12). Additionally, the Report of the 19th Communist Party of China National Congress made a strong case for expediting the equalization of basic public services and implementing the Healthy China agenda. As demand for basic public health care increases among people, China gradually increases investment in basic public health services. The standard for NEPHS per capita subsidy was increased to 79 yuan in 2021 (13). Currently, basic public health services consist mostly of 14 service components, such as health record establishment. In actuality, national primary health care services are based on the notion of voluntary participation. Effective evaluation of the project's health impact lays the groundwork for continued development and optimization of the NEPHS implementation process.

Although it has been some years since migrants received equitable access to basic public health services. However, existing research indicates that there are still issues such as underutilization and disparity in the use of NEPHS by migrants in inflow areas (14). Numerous studies have been conducted to determine the state of affairs and the factors that influence the floating population's use of specific NEPHS projects. Furthermore, these studies consistently demonstrated that migrants' overall utilization of NEPHS remains low (15–19). Analyses of specific regions have yielded similar conclusions (20–23). Additionally, research have been conducted on certain subgroups of migrants, including those with hypertension and type 2 diabetes, the elderly, and the younger generation. It was also discovered that patients with chronic diseases had a poor level of utilization of NEPHS items such as follow-up evaluation, establishment of health records, physical examination, and health education (24, 25). The elderly and young migrants also have a low degree of utilization of NEPHS (26–29).

Some studies discovered significant differences in NEPHS utilization between local residents and migrants when comparing the two groups (30). The proportion of migrants with urban household registration who establish health records is significantly greater than that of migrants with rural household registration (31). While numerous studies have examined the determinants of NEPHS utilization among migrants, few have examined the health benefits associated with increased awareness and utilization of NEPHS among residents, particularly migrants. Local residents' surveys indicated that the NEPHS project can significantly improve hypertension treatment and control (32). Maternal and child healthcare utilization and outcomes have improved markedly (33). NEPHS implementation improved hypertension and diabetes control, as well as the level of health management in patients with severe mental disorders and children (34). Additionally, NEHS has the potential to close the health disparity between residents by increasing health literacy and influencing poor residents' health-related behavior (35). Furthermore, it can help migrants access healthcare (36).

There are only two studies that we are aware of that examine the effects of NEPHS on migrant health. Both of them, however, use the fact that the city implements NEPHS as an explanatory variable in order to examine the impact on the health of migrants (37, 38). Variables constructed at the city level may not accurately reflect the details of NEPHS utilization by migrants, introducing estimation bias into estimates of the health effect. This paper examined the current state of awareness and utilization of NEPHS among Chinese migrants using data from the 2017 China Migrants Dynamic Survey (CMDS) conducted by the National Health Commission. We examined the effect of NEPHS awareness and utilization on population health, which is one of the contributions to existing research; Additionally, there may be a difficulty in studying the aforementioned effect. Migrants who live closer to a community health facility, those who have chronic diseases, and those who have had recent illnesses or injuries are more likely to use NEPHS. Thus, a direct comparison of the health disparity between migrants who use NEPHS and those who do not may lead to the conclusion that NEPHS worsens migrants' health. As a result, the estimation is skewed. Therefore, the estimation is skewed. As a result, this study controls for the above variables in the benchmark regression on the one hand and uses propensity score matching to identify the net effect of NEPHS on the health of migrants on the other hand, which is another contribution of this paper. Finally, in order to examine the distinct effects of NEPHS awareness and utilization on the health of various groups, heterogeneity analysis was used to accurately quantify the impact of NEPHS on the health of various floating populations.

## Conceptual Framework

**Figure 1:**
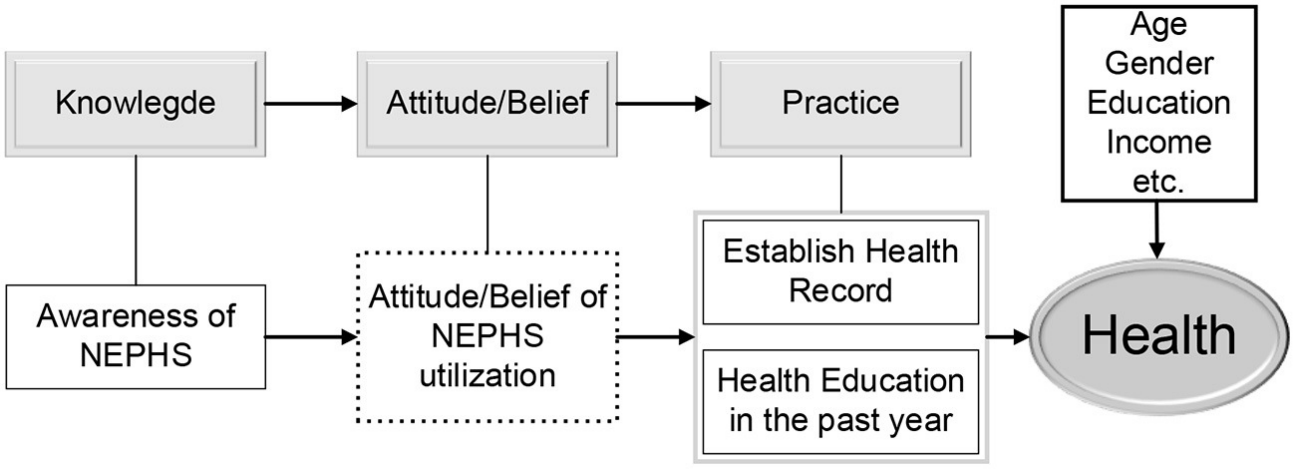
Conceptual framework.

Knowledge, Attitude / Belief, and Practice Theory (KABP) is the most prevalent model used to guide and explain how knowledge and beliefs influence health behavior change (39). According to this theory, health care knowledge and information are the foundation for building positive and accurate beliefs and attitudes, consequently altering health-related behaviors, which can improve an individual's health. Figure 1 depicts the study's conceptual framework. Clearly, information is the first step in altering an individual's behavior. Only if the floating population has a certain awareness of NEPHS will they be more likely to establish the correct health concepts and attitudes and effectively increase the health level of them. Importantly, it is only possible to utilize the related NEPHS services if they are understood beforehand. By getting health education and establishing health records, individuals can increase their focus on health management and health behavior modification, which has a substantial positive effect on their own health. Based on the preceding analysis, the following hypotheses are proposed:

Hypothesis 1: The awareness of NEPHS can effectively increase the health level of individuals, and the floating population whose health records are established and who receives health education has a higher health level. Awareness of NEPHS can promote the establishment of health records and the potential of receiving health education, so contributing to the enhancement of the health status of the floating population.

It is important to note that NEPHS may influence the self-rated health of the floating population with varying features in a way that results in differences in self-rated health. From the standpoint of age, the risk of illness will increase as persons age, and elderly usually pay greater attention to their health. Therefore, NEPHS has a greater impact on the health state of the elderly than on that of the young. In addition, as a special group, NEPHS provides once-yearly health management services for the aged, such as lifestyle and health assessment, physical examination, auxiliary examination, and health counseling. Consequently, it is likely that the health improvement effects of NEPHS will be more pronounced among the older floating population. From gender perspective, there are intrinsic differences in the health condition of men and women, with women paying greater attention to their health status when they are ill and being more ready to seek assistance for illness prevention (40). Consequently, women are more likely to be impacted by NEPHS. From the perspective of education, people with a higher level of education tend to have higher health literacy and greater health knowledge, so they have greater advantages in self-health management, but their health is less affected by NEPHS for the same reason; In addition, urban areas have more medical resources than rural areas, and there are also differences in the allocation of health human resources between urban and rural areas. Urban floating population enjoys greater accessibility and quality of medical care, thus we anticipate that the health improvement effect of NEPHS will be greater in urban regions. Based on the preceding analysis, we suggest the second hypothesis:

Hypothesis 2: Due to diverse characteristics, the impact of NEPHS awareness and utilization on the health of floating populations is heterogeneous. Specifically, NEPHS has a greater impact on the health improvement of middle-aged and elderly, women, urban floating population with low levels of education.

## Materials and Methods

### Data Source

The data for this study came from the National Health Commission's 2017 China Migrants Dynamic Survey (CMDS). The National Health Commission conducted the survey (formerly the National Health and Family Planning Commission). Sample points were chosen randomly from areas where the floating population was concentrated in 31 provinces (autonomous regions and municipalities) and the Xinjiang Production and Construction Corps. The stratified, multistage, and probability proportional to size sampling method was used to investigate migrants aged 15 and over who had lived in the inflow places for at least 1 month but were not district residents (county or city). The survey collects data on family members, household income and expenditure, employment status, mobility, and healthcare utilization, among other things. In 2017, a total of 169,989 valid samples of the floating population were collected. Because NEPHS items are primarily distributed to residents who have lived in the area for more than 6 months, samples of residents who have lived in the area for <6 months were excluded from this study. For inclusion analysis, the final valid sample size was 152,695.

### Measures

#### Health Outcomes

Self-reported health status was used as a proxy for individual health. Each respondent was asked in the CMDS, “How do you feel about your health currently?” 1 = healthy, 2 = basically healthy, 3 = unhealthy, but able to take care of themselves, 4 = unable to take care of themselves.

#### National Essential Public Health Service

##### Awareness

Each respondent to the CMDS survey was asked if they had heard of the National Essential Public Health Service. This question has two possible answers: 1 indicates yes, and 0 indicates no.

##### Utilization

In terms of NEPHS utilization, this study referred to previous research (15) and used two binary variables to determine NEPHS utilization: whether to establish health records and whether to receive any health education in the previous year. Respondents were asked whether local health records had been established. There are four options available: 1 indicates that it is established, 2 indicates that it is not established and that it has not been heard of, 3 indicates that it is not established but has been heard of, and 4 indicates that it is unclear. We unified recoded 2, 3, and 4 as 0, indicating that the individual did not establish health records in the local area. Each respondent was asked in turn if they had received health education in the following areas: occupational disease prevention, sexually transmitted diseases/AIDS prevention, reproductive health and contraception, tuberculosis prevention and control, smoking control, mental health, chronic disease prevention and control, maternal and child healthcare/healthy birth, and self-rescue in public emergencies. We combined the binary variables above and coded respondents as 1 if they had received at least one of the health education activities and 0 if they had received none.

#### Covariates

In accordance with previous studies (38, 41), this study included additional variables affecting the health of migrants in the model. Individual demographic characteristics (gender, age, ethnic minorities, marital status, education level, family size, and household registration), socioeconomic status (including household income, health insurance, and employment status), flow range, presence of chronic diseases in the past year, presence of any disease or injury in the past year, and time to the nearest health facilities are all included. Among them, marital status is a dichotomous variable; 0 indicates that the respondent is single, divorced, or widowed, while 1 indicates that the respondent is married for the first time, remarried, or cohabiting. Education level is a triadic variable, with 1 indicating primary school or less, 2 indicating junior high school, and 3 indicating senior high school or more. Household registration is a binary variable, with 1 indicating rural household registration and 0 indicating urban household registration. The model contained five dummy variables that indicated whether interviewees had participated in the New Cooperative Medical System (NCMS), the Coordinating of Urban and Rural Basic Medical Insurance (CURBMI), the Urban Resident Basic Medical Insurance (URBMI), the Urban Employee Basic Medical Insurance (UEBMI), or Free Medical Care (FMC). When assessing individual employment, we incorporate variables representing individual employment status into the model. These variables are classified as follows: 1 = unemployed, 2 = employed, 3 = employer, 4 = self-employed worker, and 5 = other. Respondents' flow range is a three-category variable, with 1 representing inter-provincial flow, 2 representing inter-city flow within the province, and 3 representing inter-county flow within the city. Respondents were asked if they had a doctor's diagnosis of high blood pressure or type 2 diabetes, with 1 indicating they had one or both and 0 indicating they did not. Similarly, respondents were asked if they had experienced any disease or injury in the preceding year, which we recoded as a binary variable, with 1 indicating yes and 0 indicating no. The time to the nearest health facility represented healthcare accessibility, which is a four-category variable: 1 equals <15 min, 2 equals 15–30 min, 3 equals 30–60 min, and 4 equals more than 60 min.

### Statistical Analyses

#### Descriptive Statistical Analysis and Difference Test

We used descriptive statistics to examine NEPHS awareness and use among Chinese migrants, and then used the chi-square test to determine whether there were significant differences in self-rated health between those who were aware of and used NEPHS and those who were unaware of and did not use NEPHS items.

#### Regression Analysis

For regression analysis, we used two models: the linear probability model (LPM) and the ordered logit model. Due to the numerous positive properties of self-rated health (42), it can be treated as a continuous variable. To facilitate comparison of propensity score matching estimate results in the following section, this study used LPM results as the benchmark. Meanwhile, our study presents the regression results for the ordered Logit model. LPM's regression model is as follows:


(1) $$Health_{i}=\alpha +\beta \cdot NEPHS_{i}+\;X_{i}^{\prime }\delta +\varepsilon_{i}$$

Where *Health*_*i*_ denotes the individual's self-rated health; *NEPHS*_*i*_ denotes whether the individual is aware of or uses the NEPHS; **X**_**i**_ denotes other covariates affecting the migrants' self-rated health; ε_*i*_ is the error term in the model. β is the coefficient effect that we are interested in, as it reflects the effect of migrants' awareness or use of NEPHS on their self-rated health. Additionally, because self-rated health is an ordered variable, we report the estimated results of ordered Logit model.

#### Propensity Score Matching

As mentioned previously, to account for the possibility of estimation bias caused by a variety of factors affecting individuals' awareness of and use of NEPHS, this study used propensity score matching to identify two groups of samples with otherwise similar characteristics in order to estimate the average treatment effect (ATT) on the health of migrants.


(2) $$\begin{array}{r} ATT=E\left[y_{1i}|D_{i}=1,P\left(X\right)\right]-E\left[y_{0i}|D_{i}=1,P\left(X\right)\right] \end{array}$$

In Equation (2), *D*_*i*_ denotes the dummy variable of *i*_*th*_ migrant's awareness or utilization of NEPHS; *D*_*i*_ = 1 indicates that the migrant was aware of or utilized NEPHS; *D*_*i*_ = 0 indicates that the migrant was unaware of or did not utilize NEPHS. *y*_1*i*_ refers to individual's self-rated health when they are aware of or use NEPHS; *y*_0*i*_ refers to the self-rated health when they are unaware or do not use NEPHS. *P*(*X*) denotes the probability that migrants are aware of or use NEPHS, also referred to as the propensity score.

For example, to examine the effect of NEPHS awareness on migrants' self-rated health, we first divide the population into those who were aware of NEPHS and those who had not heard of it. The two groups of samples were then matched using four PSM matching strategies (nearest neighbor matching, radius matching, kernel matching, and local linear regression matching). Finally, the treatment group's and control group's self-reported health scores were obtained, as well as the differences between the two groups. A similar approach was used to examine the effect of health education and the establishment of health records on migrants' self-rated health.

#### Mediating Effect Model

To further test the hypothesis of the mediating effect proposed in the conceptual framework, namely, that the awareness of NEPHS can improve the self-rated health of the floating population by increasing the likelihood of establishing health records and receiving health education in the previous year, we developed the mediating effect model (43). Analysis of the mediating impact has been widely utilized as the primary tool for testing the mechanism (44). The most popular method for confirming the mediating effect is the stepwise regression test. It is a technique for determining the existence of a mediating effect by creating three regression models and evaluating the magnitude and significance of the coefficients of key variables. This study employs the same methodology. Three regression models comprise the stepwise regression test:


(3) $$Health_{i}=\alpha_{1}+\beta_{1}\cdot Awareness_{i}+X_{i}^{\prime }\delta +\varepsilon_{i}$$

(4) $$M_{i}=\alpha_{2}+\beta_{2}\cdot Awareness_{i}+X_{i}^{\prime }\xi +\mu_{i}$$

(5) $$Health_{i}=\alpha_{3}+\beta_{3}\cdot Awareness_{i}+\gamma \cdot M_{i}+X_{i}^{\prime }\eta +\upsilon_{i}$$

Among them, *Health*_*i*_ refers to the health of the floating population, *Awareness*_*i*_ to the dummy variable representing NEPHS awareness, and **X**_**i**_ to the other covariates. The mediating variable is *M*_*i*_. In this study, the variables that serve as mediators are the establishment of health records and the receipt of health education during the past year. The coefficient β_1_ in Equation (3) represents the total effect of NEPHS awareness on health, the coefficient β_2_ in Equation (4) represents the effect of NEPHS awareness on mediating variables, and the coefficient γ in Equation (5) represents the effect of mediating variables on the health of the floating population after controlling for Awareness. When β_1_ is significant, it is possible to verify the statistical significance of 2 and. If both variables are significant, then the mediating effect is significant.

## Results

### Characteristics of the Study Population

As shown in Table 1, the average age of migrants is 37.10 ± 11.00 years; 48.5% are female; 83.75% are married; and 9.19% are members of minority groups. The majority of migrants have completed junior middle school or less, and the proportion of migrants living in rural areas is even higher, at 82.16%. The study population's annual household income per capita was 30360.10 ± 25748.25 yuan, and employees accounted for 46.68%, the highest rate. NCMS, CURBMI, URBMI, UEBMI, and FMC participation rates were 62.30, 4.81, 7.23, 22.28, and 2.19%, respectively. According to the floating range, the majority of migrants are interprovincial, followed by intercity, and intercounty, with 48.47, 33.42, and 18.11%, respectively. Additionally, 5.74% of them had been diagnosed with hypertension or type 2 diabetes by a physician, and 49.14% had suffered from diseases or injuries in the previous year. In terms of healthcare accessibility, the majority of migrants live <15 min from the nearest health facility (83.51%).

**Table 1:** Descriptive statistics of variables.

	**Total**
	**(*N* = 152,695)**
Age	
Mean (SD)	37.10 (11.00)
Family size	
Mean (SD)	3.18 (1.18)
Annual household income per capita (Yuan)	
Mean (SD)	30360.10 (25748.25)
Self-rated health	
Healthy	124,684 (81.66%)
Basically healthy	23,665 (15.50%)
Not healthy, but able to take care of oneself	4,187 (2.74%)
Unable to take care of oneself	159 (0.10%)
NCMS	
No	57,565 (37.70%)
Yes	95,130 (62.30%)
CURBMI	
No	145,351 (95.19%)
Yes	7,344 (4.81%)
URBMI	
No	141,649 (92.77%)
Yes	11,046 (7.23%)
UEBMI	
No	118,673 (77.72%)
Yes	34,022 (22.28%)
FMC	
No	149,348 (97.81%)
Yes	3,347 (2.19%)
Gender	
Male	78,631 (51.50%)
Female	74,064 (48.50%)
National minority	
No	138,662 (90.81%)
Yes	14,033 (9.19%)
Education level	
Elementary and less	26,161 (17.13%)
Junior high school	66,339 (43.45%)
Senior and above	60,195 (39.42%)
Marital status	
Unmarried	24,816 (16.25%)
Married	127,879 (83.75%)
Floating range	
Interprovincial	74,005 (48.47%)
Intercity	51,032 (33.42%)
Intercounty	27,658 (18.11%)
Hukou	
Urban	27,235 (17.84%)
Rural	125,460 (82.16%)
Chronic diseases	
Without	143,933 (94.26%)
With	8,762 (5.74%)
Sickness or injury in the past year	
No	77,661 (50.86%)
Yes	75,034 (49.14%)
Employment status	
Unemployed	27,576 (18.06%)
Employee	71,273 (46.68%)
Employer	7,560 (4.95%)
Self-employed	43,621 (28.57%)
Others	2,665 (1.75%)
Time to the nearest health facility	
within 15 min	127,519 (83.51%)
15–30 min	22,147 (14.50%)
30–60 min	2,647 (1.73%)
More than 1 h	382 (0.25%)

### Awareness and Utilization of NEPHS

**Figure 2:**
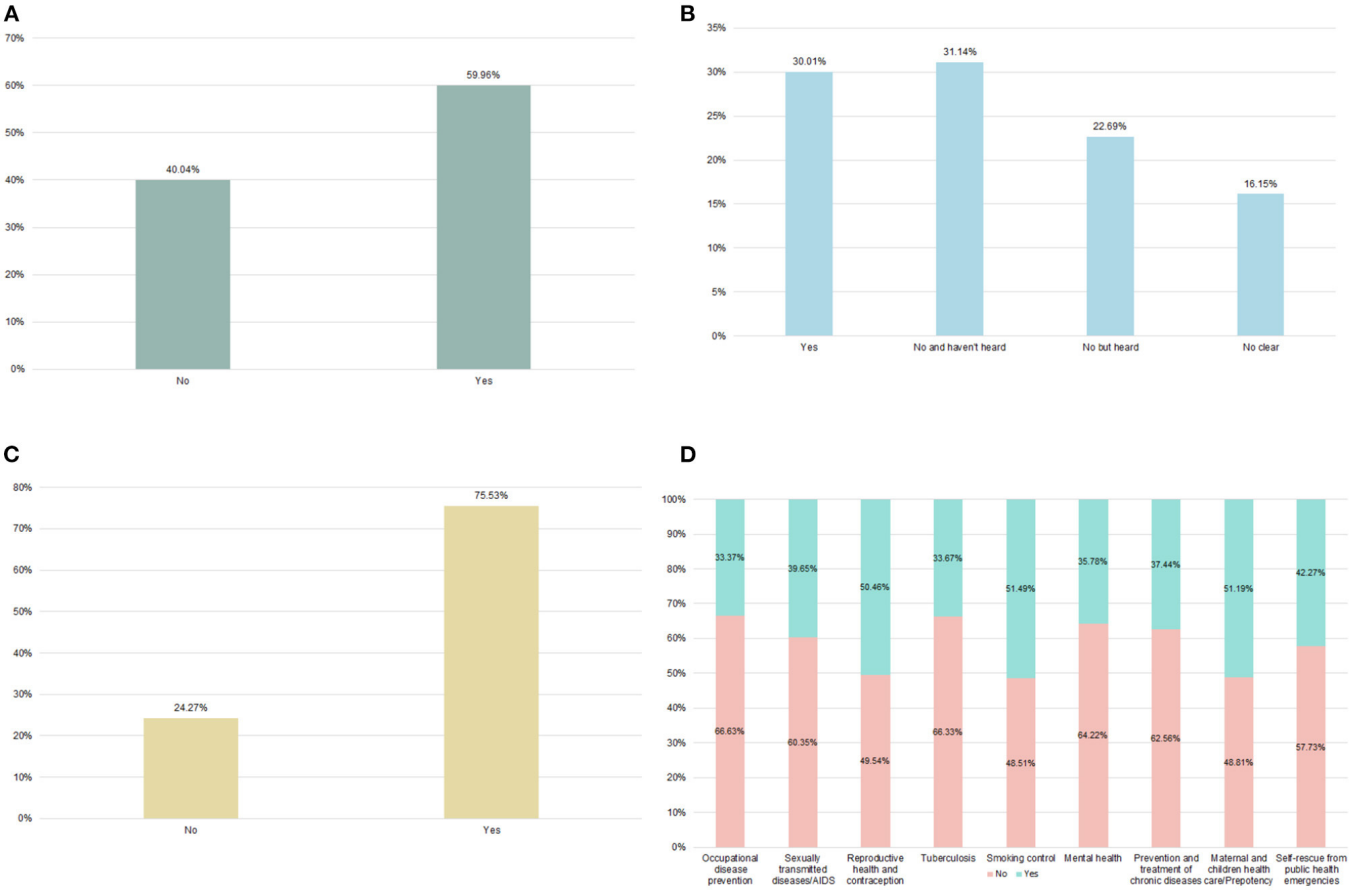
Awareness and utilization of national essential public health services (2017). **(A)** Awareness of NEPHS **(B)** Established health record **(C)** Accepted health education in past year **(D)** The health education participation rate of different types.

Figure 2 depicted Chinese migrants' specific awareness and use of NEPHS. 59.96% of migrants are aware of the NEPHS, while more than 40% have never heard of it. 30.01% of the population has established health records, while 22.69% of the population has not established health records but has heard about them. 31.14% of migrants who have not established health records and have not been informed about them, and 16.15% of the general population who are unsure whether they have established health records. 75.53% of the population has received some form of health education, while 24.27% has received no form of health education. The participation rate of migrants in each health education activity was depicted in Figure 2D. As can be seen, each health education program has a participation rate of <50%. Occupational disease and tuberculosis prevention education have the lowest participation rates, at 33.37 and 33.67%, respectively.

### Health Status Among Different Subgroups of Awareness and Utilization of NEPHS

The differences in self-rated health between those who heard about and used NEPHS and those who did not are shown in Table 2. The proportion of healthy migrants who have heard of NEPHS is significantly higher than those who have not (83.03 vs. 79.55%). The proportion of those who are basically healthy, unhealthy and unable to take care of themselves was lower among those who are aware of NEPHS (14.63 vs. 16.83%, 2.27 vs. 3.46%, 0.07 vs. 0.16%). The chi-square test revealed a significant difference in self-rated health between those aware of NEPHS and those who were not (*P* < 0.01). Similar findings were discovered when it came to health education and the establishment of a health record.

**Table 2:** Self-rated health among different subgroups of awareness and utilization of NEPHS.

**Variables**	**Healthy** **(*N* = 124,684)**	**Basically healthy** **(*N* = 23,665)**	**Not healthy, but able to** **take care of oneself** **(*N* = 4,187)**	**Unable to take care of oneself** **(*N* = 159)**	**χ^2^**
Awareness of NEPHS					
No	47,993 (79.55%)	10,156 (16.83%)	2,087 (3.46%)	94 (0.16%)	381.64^***^
Yes	76,691 (83.03%)	13,509 (14.63%)	2,100 (2.27%)	65 (0.07%)	
Accepted health education in past year					
No	32,522 (79.19%)	6,856 (16.69%)	1,607 (3.91%)	83 (0.20%)	426.35^***^
Yes	92,162 (82.56%)	16,809 (15.06%)	2,580 (2.31%)	76 (0.07%)	
Established health record					
No	86,576 (81.05%)	17,118 (16.02%)	3,009 (2.82%)	119 (0.11%)	88.80^***^
Yes	38,108 (83.07%)	6,547 (14.27%)	1,178 (2.57%)	40 (0.09%)	

*The table reported the frequency and corresponding row percentages for each variable;*

*** *P < 0.01*.

### Regression Analyses Results

LPM and ordered logit model were used to investigate the effect of NEPHS awareness and utilization on migrants' self-rated health, Table 3 summarizes the estimation results for Models 1–3, which used linear probability model, and Models 4–6, which used non-linear ordered logit model. Regardless of the LPM or Ordered Logit model used, after adjusting for the province fixed effect and other covariates, self-rated health was significantly better for migrants who had heard about NEPHS, received at least one type of health education in the preceding year, and established health records in their place of residence. All of the above effects were statistically significant at the 1% level (*P* < 0.01). The LPM findings indicated that awareness of NEPHS could improve migrants' self-rated health by ~0.038 units on average, and that receiving at least one type of health education in the previous year could improve migrants' self-rated health by ~0.03 units. Similarly, establishing a health record in the residence area increases self-rated health by an average of 0.035 units.

**Table 3:** The impact of awareness and utilization of EPHS on health status of migrants.

**Variables**	**Model 1**	**Model2**	**Model 3**	**Model 4**	**Model 5**	**Model 6**
Awareness of NEPHS (Ref: No)	−0.038^***^ (0.003)			−0.241^***^ (0.022)		
Health education in the past year (Ref: No)		−0.030^***^ (0.004)			−0.172^***^ (0.026)	
Established health record (Ref: No)			−0.035^***^ (0.004)			−0.249^***^ (0.027)
Covariates	Yes	Yes	Yes	Yes	Yes	Yes
Province	Yes	Yes	Yes	Yes	Yes	Yes
Adjusted R-squared/ Pseudo R-squared	0.200	0.199	0.200	0.148	0.147	0.148

*Robust Standard errors clustered in province level are reported in the Parentheses:*

*** *P < 0.01*.

### Robust Test: Propensity Score Matching Estimates Results

Although we controlled for as many covariates as possible that could introduce bias into the benchmark regression, we used propensity score matching to determine the homogeneous individuals between those who knew or used NEPHS and those who did not. By comparing the self-reported health of two groups of homogeneous migrants, the average effect of NEPHS on the migrants' self-reported health could be determined.

According to the results of propensity score matching (Table 4), awareness of NEPHS, having received at least one health education in the preceding year, and maintaining health records could significantly improve migrants' self-rated health (*P* < 0.01). NEPHS awareness would improve migrants' self-rated health by ~0.03–0.039 units on average. Receiving health education has been shown to improve the population's self-rated health by ~0.022–0.025 units. Establishing health records could improve the floating population's self-rated health by an average of 0.028–0.031 units.

**Table 4:** PSM estimation results.

**Variables**	**Matching method**	**Treatment group**	**Control group**	**ATT**	**Bootstrap standard error**
Awareness of NEPHS	Nearest neighbor matching	1.194	1.224	−0.030^***^	0.004
	Radius matching	1.194	1.228	−0.034^***^	0.003
	Kernel matching	1.194	1.228	−0.034^***^	0.003
	Locally linear matching	1.194	1.233	−0.039^***^	0.004
Health education in the past year	Nearest neighbor matching	1.192	1.214	−0.022^***^	0.004
	Radius matching	1.192	1.214	−0.022^***^	0.003
	Kernel matching	1.192	1.216	−0.024^***^	0.004
	Locally linear matching	1.192	1.217	−0.025^***^	0.004
Established health record	Nearest neighbor matching	1.197	1.225	−0.028^***^	0.004
	Radius matching	1.197	1.227	−0.031^***^	0.003
	Kernel matching	1.197	1.226	−0.030^***^	0.003
	Locally linear matching	1.197	1.227	−0.031^***^	0.004

*Standard errors of PSM are obtained by the Bootstrap with a sample size of 500;*

*** *P < 0.01*.

### Heterogeneity Analysis

Given that the effects of NEPHS on migrants are likely to be influenced by their age, gender, level of education, and household registration, the study samples were further divided into subgroups based on four variables mentioned above. As before, similar LPM regressions were conducted, and Table 5 displayed the corresponding estimation results. The NEPHS had significantly different effects on the self-rated health of different subgroups of migrants. Specifically, awareness of the NEPHS, recent health education, and the establishment of health records had a greater impact on the self-rated health of migrants over 45 years old, particularly the elderly, and a relatively smaller impact on the health of migrants aged 15–44 years old. In terms of gender, the three NEPHS variables have a greater effect on the health of female migrants than on male migrants. Migrants with a primary education level or less were more likely to have their self-rated health influenced by NEPHS. Additionally, NEPHS improved urban migrants' health more than rural migrants' health.

**Table 5:** Heterogeneity analysis of the impact of NEPHS on health status.

**Variables**	**By age**			**By gender**	
	**15–44**	**45–59**	**Above 60**	**Male**	**Female**
Awareness of NEPHS	−0.030^***^ (0.003)	−0.060^***^ (0.008)	−0.058^***^ (0.019)	−0.034^***^ (0.004)	−0.042^***^ (0.004)
Health education in the past year	−0.020^***^ (0.004)	−0.042^***^ (0.008)	−0.072^***^ (0.021)	−0.023^***^ (0.004)	−0.037^***^ (0.006)
Established health record	−0.033^***^ (0.004)	−0.042^***^ (0.008)	−0.046^**^ (0.021)	−0.029^***^ (0.005)	−0.041^***^ (0.005)
**Variables**	**By education level**			**By residence registration**	
	**Elementary school and below**	**Junior school**	**Senior and above**	**Urban**	**Rural**
Awareness of NEPHS	−0.054^***^ (0.009)	−0.037^***^ (0.004)	−0.032^***^ (0.004)	−0.040^***^ (0.006)	−0.038^***^ (0.004)
Health education in the past year	−0.043^***^ (0.010)	−0.031^***^ (0.005)	−0.022^***^ (0.004)	−0.036^***^ (0.007)	−0.029^***^ (0.004)
Established health record	−0.047^***^ (0.010)	−0.037^***^ (0.005)	−0.029^***^ (0.004)	−0.038^***^ (0.007)	−0.035^***^ (0.004)

*Robust Standard errors clustered in province level are reported in the Parentheses:*

*** *P < 0.01*.

### Mechanism Analysis

To validate the mechanism of NEPHS on the health of the floating population, we developed a model of the mediating effect. In Table 3, we have established Equation (3) for the model of the mediating effect. Here, we estimated Equations (4) and (5) using the establishment of health records and receiving health education in the previous year as the mediating variables, respectively. Table 6 displayed the estimation results. Model 1 in Table 3 represented the effect of NEPHS awareness on the self-rated health of the floating population, which is denoted by the coefficient β_1_ in Equation (3). In Table 6, Model 1 illustrated the impact of NEPHS awareness on the establishment of health record. It is evident that NEPHS awareness could significantly increase the possibility of establishing health record for the floating population (*P* < 0.01). Model 2 estimated the impact of NEPHS awareness on health after controlling the establishment of health record variable and which was statistically significant at the 1% level (*P* < 0.01). NEPHS awareness still had a favorable influence on the self-rated health of the floating population. Similarly, awareness of NEPHS might considerably improve the likelihood that the floating population has received health education in the previous year (*P* < 0.01). Awareness of NEPHS remains to have a beneficial influence on the self-rated health of the floating population when the mediating variable is included in the model (*P* < 0.01).

**Table 6:** The results of mediating effect model.

	**Model 1**	**Model 2**	**Model 3**	**Model 4**
**Variables**	**Established** **health** **record**	**Self-rated** **health**	**Accepted** **health** **education in** **the past year**	**Self-rated** **health**
Awareness of NEPHS	0.361^***^ (0.010)	−0.030^***^ (0.003)	0.267^***^ (0.007)	−0.033^***^ (0.003)
Established health record		−0.022^***^ (0.004)		
Accepted health education in the past year				−0.019^***^ (0.004)
Covariates	Yes	Yes	Yes	Yes
Province	Yes	Yes	Yes	Yes
Adjusted R-squared	0.238	0.200	0.162	0.200

*Robust Standard errors clustered in province level are reported in the Parentheses:*

*** *P < 0.01*.

## Discussion

China has proposed the goal of equalization of national essential public health services for the population since 2013 in order to improve the health status of migrants. Due to the mobility characteristics of the population, it is difficult to conduct NEPHS. The CMDS survey data from 2017 were analyzed in this study to determine the effect of NEPHS awareness or utilization on the health of migrants. Analyses of the current state of awareness and utilization of NEPHS could be used to target areas for improvement in NEPHS implementation. Further investigation of the floating population's health effects and heterogeneity enables evaluation of the NEPHS project's effectiveness, clarification of the NEPHS project's primary improvement direction, and effective improvement of the floating population's health and welfare.

NEPHS's mission is to increase access to and equity in essential public health services. However, our study found that migrants' awareness and utilization of NEPHS are still insufficient, impeding health equity. This is consistent with previous research findings (15, 45, 46). Although the NEPHS project has been in operation for more than a decade, public awareness and utilization of basic public health services remain low. According to a survey conducted in some regions of China, only 0.2 percent of the surveyed floating population was familiar with the entire content of NEPHS items, and there was also an issue of unbalanced development across service items (46). The possible reason is that the floating population's mobility makes it difficult to implement the NEPHS strictly, and the migrants' coverage rate for health records, health education, and health examinations, as well as other services, is mediocre (14). Migrants' ability and initiative to obtain relevant NEPHS information is less developed than that of local residents (23, 47). Simultaneously, the unbalanced allocation of health resources and a lack of human resources for NEPHS contribute to migrants' low awareness and utilization of NEPHS (16, 45).

After adjusting for other covariates, our study demonstrated that increased awareness and use of NEPHS have a beneficial effect on the health of migrants. Our study also indicated that the awareness of NEPHS will promote the establishment of health record of floating population and the likelihood of receiving health education in the last year, consequently increasing their health status. According to the KABP model, the awareness of NEPHS would increase the floating population' s cognition and comprehension of public health services, and then promote them to gradually build a belief that is favorable to their own health. Finally, this positive belief and attitude can be turned into healthy behaviors. By establishing health records and receiving health education, the health literacy of the floating population can be effectively increased, so that individuals attach importance to the monitoring and management of self-health, and thus have a beneficial impact on the health of the floating population.

To a certain extent, awareness of NEPHS reflects the population's health literacy. Several studies found that equalizing NEPHS among migrants *via* a quasi-natural experiment found that equalizing NEPHS could improve the population's health literacy (38). Individuals with a higher level of health literacy have a greater likelihood of being in better health (48). Improving migrants' health literacy increased their chances of establishing health records and receiving additional health education, which had a positive effect on their use of NEPHS (49). This increase in healthcare utilization would also have a positive effect on health outcomes (50). The establishment of health records can significantly increase awareness of individual health management (45), and also assist health facilities and personnel in monitoring the health of migrants on a regular basis. As a result, it is critical for improving the population's health outcomes. It is self-evident that health education benefits migrants' health. Health education assists the floating population in developing a healthy lifestyle and behavior, and increasing their sense of self-efficacy for behavior change, thereby facilitating their health level improvement (51).

Additionally, our study discovered significant differences in the health effects of NEPHS awareness and utilization across subgroups. The effect of increased awareness and use of NEPHS on health is greater for middle-aged and elderly people, women, and low-educated migrants with urban household registration. Wang et al. found that women establish health records at a higher rate than men, and older immigrants establish health records at a higher rate than younger migrants due to their increased risk of chronic diseases. Additionally, the elderly and women are NEPHS's primary target groups (45). Simultaneously, due to their increased health risks, women and the elderly are more concerned about their own health (52). As a result, the health benefits associated with increased awareness and use of NEPHS are greater for women and elderly migrants. Individuals with a higher education degree are more likely to use NEPHS, which is consistent with our study conclusion (45). By and large, those with a higher level of education have a higher level of health literacy, which means they pay more attention to their health. However, they also have greater access to health knowledge as a result of their higher education level. As a result, NEPHS have a lower effectiveness in improving their health. Additionally, while China is currently reforming its household registration system in order to eliminate social welfare disparities between urban and rural residents, the household registration system continues to have an effect on how urban and rural residents use NEPHS (45). Residents of rural areas have a lower awareness of NEPHS than residents of urban areas (34). Urban migrants, in comparison to rural migrants, are more likely to establish health records (31, 45, 53). Furthermore, NEPHS's health-improving effect is severely limited by a shortage of professional talent and an unbalanced structure of primary health care facilities in rural areas (54).

This study has policy implications, first and foremost, because migrants' awareness of NEPHS is still low, and existing public health publicity methods have been unable to meet resident demand. As a result, a variety of new media communication channels, such as WeChat and other new media, were required to increase the visibility and reach of NEPHS, to fully exploit the subjective initiative in utilizing NEPHS, and to improve NEPHS utilization; Second, health education and public awareness about health issues should be bolstered for migrants. Health administration departments should conduct low-participation health education activities, such as occupational disease and tuberculosis prevention, and utilize information technology to establish health records and fully utilize electronic health records in order to achieve dynamic health management of migrants within the local community. Finally, given the heterogeneity of NEPHS's health effects and the individual characteristics of migrants, targeted NEPHS projects should be conducted for specific groups, such as men, young and middle-aged people, those with a high level of education, and rural migrants, in order to maximize the health benefits of NEPHS.

Additionally, our study has some limitations. Based on the cross nature of the CMDS data, we are unable to accurately determine the long-term effect of NEPHS on health among migrants. Second, due to variable limitations in the data, we are unable to verify the specific mechanism by which NEPHS utilization affects health. Numerous studies have established a link between NEPHS use and health literacy. Our future research will examine whether the use of NEPHS can help individuals improve their health literacy and develop healthy behaviors, thereby improving their health outcomes.

## Conclusions

The purpose of this study was to determine the level of awareness and utilization of NEPHS among Chinese migrants and to assess their health-improving effect. The findings indicated that, despite the fact that NEPHS has been in place for over a decade, awareness and utilization of NEPHS remain low among migrants. NEPHS awareness and use had a significant positive effect on the health of migrants. The awareness of NEPHS could promote its utilization and further improve the health status of floating population. However, there are significant differences in the health effects of NEPHS awareness and use across subgroups. During the implementation of NEPHS, targeted measures such as increasing NEPHS publicity efforts and scope, conducting health education activities with a low participation rate, and focusing on males, young and middle-aged adults, those with a high level of education, and rural migrants should be taken.

## Data Availability Statement

The data analyzed in this study is subject to the following licenses/restrictions: The datasets employed in our study are not readily available because the data is provided by the Migrant Population Service Center, National Health Commission P.R. China and we have signed a legally binding agreement with the institution that we would not share any original data to any third parties. Requests to access these datasets should be directed to XX, xuxinpeng@njmu.edu.cn.

## Ethics Statement

Ethical approval for the study was not required since it was based exclusively on the publicly available data, CMDS. Hence the study subjects were not directly approached.

## Author Contributions

XX and HY designed the study. QZ and XX led the data analysis and wrote the manuscript. HY, QZ, XX, and QW participated in the revision of the manuscript and approved the final version for publication.

## Funding

This study was supported by the Open Project of Adverse Drug Reaction Monitoring Center of Family Planning Drugs of National Health Commission/Jiangsu Health Development Research Center (JSHD2021050), Cultivation Project of Decision-making Consultation, Institute of Healthy Jiangsu Development, Nanjing Medical University (7).

## Conflict of Interest

The authors declare that the research was conducted in the absence of any commercial or financial relationships that could be construed as a potential conflict of interest.

## Publisher's Note

All claims expressed in this article are solely those of the authors and do not necessarily represent those of their affiliated organizations, or those of the publisher, the editors and the reviewers. Any product that may be evaluated in this article, or claim that may be made by its manufacturer, is not guaranteed or endorsed by the publisher.

## Abbreviations

NEPHS: National Essential Public Health Service
CMDS: China Migrants Dynamic Survey
NCMS: New Cooperative Medical System
CURBMI: Coordinating of Urban and Rural Basic Medical Insurance
URBMI: Urban Resident Basic Medical Insurance
UEBMI: Urban Employee Basic Medical Insurance
FMC: Free Medical Care.
